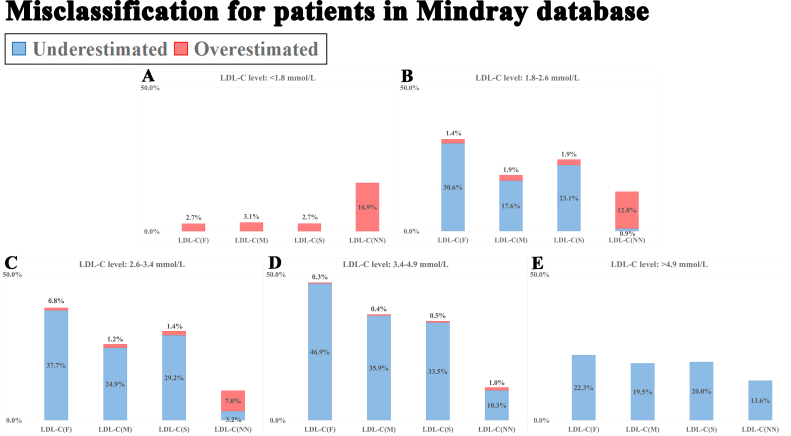# Neural network model outperforms conventional equations in LDL cholesterol estimation: A comparative study of 188,887 Chinese individuals with focus on hypertriglyceridemia

**DOI:** 10.1016/j.athplu.2025.11.003

**Published:** 2025-11-26

**Authors:** Hao Xue, Yu Lin, Youlin Liu, Lin Li, Pengzhen Chen, Mingyang Li, Ling Ji, Yong Xia

**Affiliations:** a Department of Laboratory Medicine, Peking University Shenzhen Hospital, Shenzhen, China; b Shenzhen Mindray Bio-Medical Electronics Co.,Ltd, China

**Keywords:** Low-density lipoprotein cholesterol, Neural network, Hypertriglyceridemia, Friedewald equation, Clinical decision

## Abstract

**Background:**

Accurate estimation of low-density lipoprotein cholesterol (LDL-C) is crucial for atherosclerotic cardiovascular disease (ASCVD) risk management. Extensive international validation studies have demonstrated that traditional formulas (Friedewald, Martin/Hopkins, Sampson) often yield significant errors under conditions of extreme hypertriglyceridemia. This study aimed to assess the performance of these conventional formulas in Chinese populations and develop a novel neural network–based LDL-C estimation model [LDL-C(NN)].

**Methods:**

In this retrospective study, we analyzed 188,887 lipid profiles—including total cholesterol, triglycerides, high-density lipoprotein cholesterol, and directly measured LDL-C—from Peking University Shenzhen Hospital using Mindray (outpatients, n = 83,731) and Beckman (inpatients, n = 105,156) systems. The test results from the two detection systems are non-overlapping. We used stratified random sampling based on TG levels to select 30,000 profiles from each of the two systems as the training dataset (60,000 profiles in total). Within this training dataset, 70 % of profiles were used for parameter learning, 15 % were used for early-stopping validation, and 15 % were used for post-training testing. The remaining profiles constituted the independent test set for the final performance evaluation (Mindray: n = 53,731; Beckman: n = 75,156). We then compared the performance of LDL-C(NN) with the Friedewald, Martin/Hopkins, and Sampson formulas using correlation coefficient (r), root mean square error (RMSE), Concordance Correlation Coefficient (CCC) and clinical risk stratification accuracy.

**Results:**

Compared with directly measured LDL-C, LDL-C(NN) demonstrated higher correlation and lower RMSE than other traditional LDL-C equations in the Mindray system (r = 0.9778, RMSE = 0.1762 mmol/L; vs Friedewald quation: r = 0.8894, RMSE = 0.4783 mmol/L; vs Martin/Hopkins quation: r = 0.9658, RMSE = 0.2463 mmol/L; vs Sampson quation: r = 0.9548, RMSE = 0.2934 mmol/L, particularly patients with high triglycerides (TG levels, 9.03–13.56 mmol/L, neural network Model: CCC = 0.8750, vs Friedewald quation: CCC = 0.3320; vs Martin/Hopkins quation: CCC = 0.7278; vs Sampson quation: CCC = 0.4176). Beckman database shows the same performance. The clinical classification accuracy for LDL-C(NN) reached 87.5 % (Mindray) and 83.4 % (Beckman), surpassing that of other traditional LDL-C equations (66.6–78.7 %).

**Conclusions:**

By overcoming the linear assumptions of conventional equations, the neural network–based model significantly improves LDL-C estimation in hypertriglyceridemia (especially≥9.03 mmol/L) and complex lipid profiles, thereby expanding the applicability of traditional formulas, while demonstrating robust performance across multiple analytical systems.

## Highlights

• First neural network LDL-C model overcomes linear formula limitations.
• Solves 120 % negative bias in existing high-TG formulas (CCC = 0.88 vs 0.28–0.66).
• Reduces high-TG misclassification by 63 % with 87.5 % clinical accuracy.
• Excel-compatible formula enables lab integration.

## Graphical abstract

**Figure:**
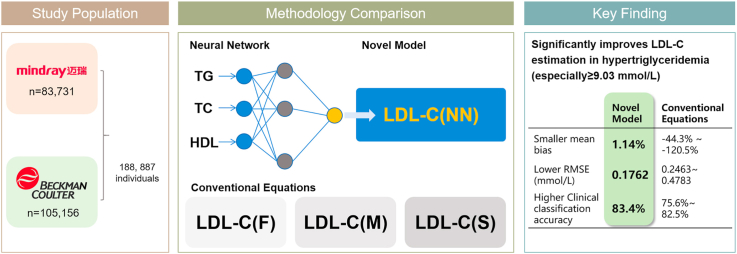

## 1 Introduction

Cardiovascular diseases (CVDs) are a leading cause of death and one of the most significant contributors to disability worldwide. According to the 2023 Global Burden of Disease (GBD) study, global CVD deaths increased from 12.31 million in 1990 to 19.2 million in 2023. Since 1990, the number of prevalent CVD cases has more than doubled, rising from 311 million globally in 1990 to 626 million in 2023. Globally, high systolic blood pressure, dietary risks, high LDL cholesterol, and air pollution were identified as modifiable risk factors contributing to the CVD burden in 2023 [1,2]. Low-density lipoprotein cholesterol (LDL-C) is a key atherogenic factor in atherosclerotic cardiovascular disease (ASCVD) and has been prioritized for risk assessment and intervention in major cardiovascular guidelines (e.g., AHA/ACC [1], ESC [3]). Despite its critical role, precise measurement of LDL-C remains challenging.

Although ultracentrifugation is the “gold standard” for LDL-C measurement, its high cost and complex operation hinder wide clinical adoption [4]. Consequently, various indirect calculation methods have become commonplace. Among these, the Friedewald formula [5], which assumes very low-density lipoprotein-cholesterol (VLDL-C) (mmol/L) = triglyceride/2.2, is widely used but frequently underestimates LDL-C at high triglyceride (TG) concentrations (≥4.52 mmol/L) or when LDL-C itself is very low (<1.8 mmol/L) [6]. In some instances, it even generates negative values, which are biologically implausible and can mislead therapeutic decisions in intensive lipid-lowering treatment.

Several modified equations, such as those by Martin/Hopkins [7] and Sampson [8], have addressed some limitations of traditional linear assumptions by introducing dynamic or polynomial coefficients. Nonetheless, these models still show considerable error in extreme hypertriglyceridemia (e.g., TG ≥ 9.03 mmol/L) [9] and continue to rely on fixed parameters within a largely linear framework. Moreover, these conventional formulas remain unvalidated in Chinese cohorts using large-scale real-world data.

Advances in artificial intelligence [10], particularly neural networks, offer new avenues to overcome the inherent limitations of these formulas. Neural networks [11] use multilayer nonlinear mapping and adaptive learning to derive hidden patterns from complex data—a capability that has already shown promise in medical imaging analysis and precision risk prediction [[12], [13], [14], [15]]. This study pioneered the groundbreaking application of neural networks to LDL-C estimation, developing a neural network based LDL-C estimation model [LDL-C(NN)] trained on large-scale datasets from multiple analytical systems. We aimed to validate the applicability of conventional formulas (Friedewald, Martin/Hopkins, Sampson) in Chinese populations using large-scale data and develop a novel neural network-based formula for LDL-C estimation.

## 2 Materials and methods

### 2.1 Study population

Between March 2023 and May 2024, we retrospectively collected a total of 188,887 lipid profiles from inpatients and outpatients of Peking University Shenzhen Hospital, using Mindray BS-2800M analyzer (outpatients, n = 83,731) and Beckman AU5800 analyzer (inpatients, n = 105,156) systems. The test results from the two detection systems are non-overlapping. Each profile contained complete results for total cholesterol (TC), triglycerides (TG), high-density lipoprotein cholesterol (HDL-C), and directly measured LDL-C (homogeneous method). The study cohort was selected based on complete availability of all four lipid parameters (TC, TG, HDL-C and LDL-C), with exclusion of specimens demonstrating missing data or invalid measurements due to technical artifacts (e.g., analytical instrument malfunction), and patients under 18 years of age were excluded.

This study was approved by the institutional review boards of Peking University Shenzhen Hospital (approval number: 2024090), which waived the requirement for informed consent due to the retrospective nature of the investigation.

### 2.2 Measurement methods and quality control

#### 2.2.1 Lipid analysis

All samples were analyzed strictly following the manufacturer's instructions and standard operating procedures using Beckman AU5800 biochemical analyzers (Beckman Coulter, USA) and Mindray BS-2800M biochemical analyzers (Mindray, CHN) with corresponding original reagents. Enzymatic methods were employed for TC detection (Beckman: OSR6216; Mindray: 105-001595-00) and TG detection (Beckman: OSR61118; Mindray: 105-001596-00), while homogeneous assays were used for HDL-C (Beckman: OSR6287; Mindray: 105-004610-00) and LDL-C (Beckman: OSR6283; Mindray: 105-004611-00) measurements. For samples exceeding the declared linearity range of reagents, appropriate dilution was performed to ensure accurate results.

All assays were performed strictly according to the manufacturers’ instructions. Both systems participated in external quality assurance programs (National Center for Clinical Laboratories, China) and employed daily internal quality control (Bio-Rad Liquichek™) to ensure analyzer reproducibility and reliability, with all lipid measurements meeting precision, bias, and total allowable error targets specified in international guidelines [16].

According to manufacturer specifications, when TG ≤ 11.3 mmol/L or Lipemia Index (L-index) ≤300 mg/dL, interference with LDL-C testing is <10 %, allowing laboratories to report LDL-C results directly. When TG > 11.3 mmol/L or L-index >300 mg/dL, samples require dilution to reduce TG/L-index within the manufacturer-stated interference-resistant concentration range, with LDL-C results reported using appropriate dilution factors.

#### 2.2.2 Data cleaning and merging

Data from both systems were compiled in a unified database after excluding cases with missing data, with the final dataset comprising 83,731 analytical profiles from the Mindray system and 105,156 from the Beckman system. High-volume data validation confirmed comparability and consistency between these analytical platforms, while standardized quality control (QC) workflows established reliability across both systems.

### 2.3 Neural network model development

#### 2.3.1 Input Variables and target

Input Variables include TC, TG, and HDL-C (all in mmol/L) and Target Output includes Directly measured LDL-C (mmol/L).

#### 2.3.2 Network architecture

We employed a feedforward neural network with a 3-10-1 structure (Fig. 1). The hidden layer consisted of 10 neurons. The neural network employs a hyperbolic tangent function as the activation function for each neuron in the network.

**Fig. 1:**
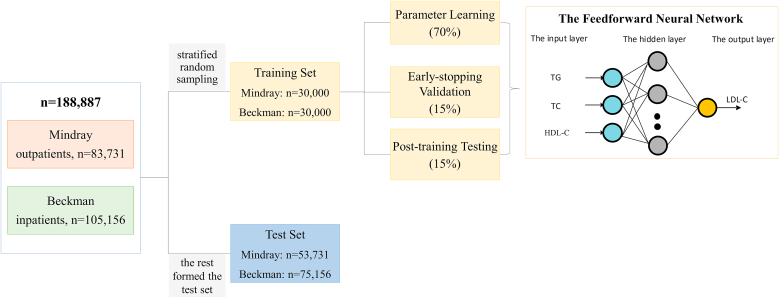
**The Feedforward Neural Network** In this retrospective study, we analyzed 188,887 lipid profiles—from Peking University Shenzhen Hospital using Mindray (outpatients, n = 83,731) and Beckman (inpatients, n = 105,156) systems. The test results from the two detection systems are non-overlapping. We used stratified random sampling based on TG levels to select 30,000 profiles from each of the two systems as the training dataset (60,000 profiles in total). Within this training dataset, 70 % of profiles were used for parameter learning, 15 % were used for early-stopping validation, and 15 % were used for post-training testing. The remaining profiles constituted the independent test set for the final performance evaluation (Mindray: n = 53,731; Beckman: n = 75,156). TC: total cholesterol; TG: triglycerides; HDL-C: high-density lipoprotein cholesterol; LDL-C: low-density lipoprotein cholesterol.

#### 2.3.3 Data splitting

To build and validate the model, we combined data from the Mindray system and the Beckman system and then used stratified random sampling based on TG levels to select 30,000 profiles from each of the two systems as the training dataset (60,000 profiles in total). Within this training dataset, 70 % of profiles were used for parameter learning, 15 % were used for early-stopping validation, and 15 % were used for post-training testing.

The remaining profiles constituted the independent test set for the final performance evaluation (Mindray: n = 53,731; Beckman: n = 75,156). The performance of the neural network model was evaluated on the Mindray and Beckman test sets respectively. This allocation ensured adequate representation of high-TG (≥9.03 mmol/L) samples in both training and test cohorts.

#### 2.3.4 Training process

We trained the network using the Neural Net Fitting toolbox in MATLAB R2023a, with mean squared error (MSE) as the loss function and the Levenberg-Marquardt algorithm for weight updates. Upon completion, the best-performing weight matrix was saved and exported as a simplified formula executable in spreadsheet software (e.g., Microsoft Excel), enabling broader clinical or research application.

### 2.4 Formula validation and performance metrics

#### 2.4.1 Traditional formulas

We compared the newly developed LDL-C(NN) model against three widely used formulas as follows: Friedewald formula [5] [LDL-C(F)], Martin/Hopkins formula [7] 240-Cell [17] [LDL-C(M)], and Sampson formula [8] [LDL-C(S)].

#### 2.4.2 Overall performance

We calculated correlation coefficient (r) and the root mean square error (RMSE) for each approach relative to directly measured LDL-C. To further examine systematic bias and agreement, we calculated Concordance Correlation Coefficient (CCC).

### 2.5 Subgroup analysis

Based on TG levels, the dataset was divided into four groups: <2.26, 2.26–9.03, 9.03–13.56, and ≥13.56 mmol/L. We compared each formula's estimation error across these subgroups to assess performance in varying lipid profiles.

#### 2.5.1 Clinical classification accuracy

Using standard LDL-C cutoffs (1.8, 2.6, 3.4, and 4.9 mmol/L) from the Chinese guidelines for lipid management (2023 edition), we assessed how accurately each formula classified patients into clinical risk categories. Misclassification rates and directions (overestimation or underestimation) were also analyzed.

#### 2.5.2 Additional analyses

To address routine laboratory evaluation methods more comprehensively, Passing-Bablok regression was conducted as a supplementary analysis. This step provided additional insight into proportional and constant biases among the different formulas.

### 2.6 Statistical analysis

All statistical analyses were performed using MedCalc 15.2.2. Normally distributed quantitative data were expressed as mean ± standard deviation (SD), while non-normally distributed data were presented as median with interquartile range (IQR, 25th-75th percentile).

## 3 Results

### 3.1 Baseline characteristics

Table 1 summarizes the overall lipid distributions obtained from the Mindray system (n = 83,731) and the Beckman system (n = 105,156), with mutually exclusive patient cohorts between the two detection systems. Use the median with interquartile range (IQR, 25th-75th percentile). The total cholesterol (TC) and LDL-C levels for the Mindray system data were 4.45 (3.81–5.11) mmol/L and 2.70 (2.15–3.32) mmol/L, respectively. For the Beckman system data, the corresponding medians were 4.90 (4.19–5.66) mmol/L for TC and 3.03 (2.49–3.61) mmol/L for LDL-C. Extremely high TG (≥9.03 mmol/L) was observed in 0.61 % of Mindray system and 0.57 % of Beckman system.

**Table 1:** Distribution of lipid results for Mindray and Beckman databases.

Database	Range	Median (25th-75th Percentile)
**Mindray (N = 83731)**		
TC (mmol/L)	0.38-19.78	4.45 (3.81–5.11)
TG (mmol/L)	0.01–29.53	1.35 (0.94–1.96)
HDL-C (mmol/L)	0.01–4.48	1.22 (0.98–1.48)
dLDL-C-MR (mmol/L)	0.13–18.35	2.70 (2.15–3.32)
LDL-C(F) (mmol/L)	(-8.49)-14.23	2.42 (1.86–3.05)
LDL-C(M) (mmol/L)	(-3.94)-15.08	2.51 (1.97–3.14)
LDL-C(S) (mmol/L)	(-0.83)-11.61	2.50 (1.93–3.13)
**Beckman (N = 105156)**		
TC (mmol/L)	0.85-34.83	4.90 (4.19–5.66)
TG (mmol/L)	0.25–110.96	1.20 (0.86–1.75)
HDL-C (mmol/L)	0.12-10.81	1.30 (1.10–1.53)
dLDL-C-B (mmol/L)	0.29-15.52	3.03 (2.49–3.61)
LDL-C(F) (mmol/L)	(-23.38)-17.75	2.91 (2.29–3.54)
LDL-C(M) (mmol/L)	(-11.85)-17.97	2.96 (2.37–3.56)
LDL-C(S) (mmol/L)	(-4.53)-29.57	2.97 (2.36–3.61)

TC, total cholesterol; TG, triglyceride; HDL-C, high-density lipoprotein cholesterol; dLDL-C-MR, directly measured LDL-C using the Mindray system; dLDL-C-B, directly measured LDL-C using the Beckman system; LDL-C(F), calculated LDL-C using the Friedewald formula; LDL-C(M), calculated LDL-C using the Martin/Hopkins formula; LDL-C(S), calculated LDL-C using the Sampson formula.

SI Conversion Factors: To convert TC, HDL-C, non-HDL-C, or LDL-C from mg/dL to mmol/L, multiply by 0.0259. To convert TG from mg/dL to mmol/L, multiply by 0.0113.

We used stratified random sampling based on TG levels to select 30,000 profiles from each of Mindray system and Beckman system as the training dataset (60,000 profiles in total). Within this training dataset, 70 % of profiles were used for parameter learning, 15 % were used for early-stopping validation, and 15 % were used for post-training testing. The remaining profiles constituted the independent test set for the final performance evaluation (Mindray: n = 53,731; Beckman: n = 75,156).

### 3.2 Overall performance of the formulas

Table 2 compares the performance of the neural network–based model [LDL-C(NN)] and traditional formulas in the independent test sets for each analytical system. LDL-C(NN) consistently demonstrated higher correlation (r), higher Concordance Correlation Coefficient (CCC) and lower root mean square error (RMSE) relative to directly measured LDL-C (Table 2).

**Table 2:** Pearson correlation coefficient, Root Mean Square Error and Concordance Correlation Coefficient between the estimated LDL-C values and the measured LDL-C values, in the overall sample and in triglycerides subclasses.

LDL-C estimation type	Mindray database					Beckman database				
	N	r	RMSE	CCC	CCC 95 % CI	N	r	RMSE	CCC	CCC 95 % CI
			(mmol/L)					(mmol/L)		
**Overall sample**										
LDL-C(F)	53731	0.8894	0.4783	0.8395	0.8372 - 0.8418	75156	0.8542	0.5606	0.8314	0.8293 - 0.8335
LDL-C(M)	53731	0.9658	0.2463	0.9471	0.9463 - 0.9479	75156	0.9445	0.3263	0.9369	0.9360 - 0.9377
LDL-C(S)	53731	0.9548	0.2934	0.9303	0.9292 - 0.9314	75156	0.9197	0.3930	0.9110	0.9098 - 0.9121
LDL-C(NN)	53731	0.9778	0.1762	0.9757	0.9753 - 0.9761	75156	0.9742	0.2379	0.9648	0.9643 - 0.9653

**TG class**										
**TG<2.26 mmol/L**										
LDL-C(F)	43944	0.9778	0.2634	0.9544	0.9537 - 0.9552	64764	0.9743	0.2287	0.9674	0.9669 - 0.9678
LDL-C(M)	43944	0.9851	0.2184	0.9682	0.9676 - 0.9687	64764	0.9824	0.1884	0.9774	0.9771 - 0.9777
LDL-C(S)	43944	0.9827	0.2159	0.9697	0.9691 - 0.9702	64764	0.9785	0.2180	0.9708	0.9704 - 0.9711
LDL-C(NN)	43944	0.9857	0.1508	0.9839	0.9836 - 0.9842	64764	0.9847	0.2052	0.9718	0.9714 - 0.9722

**2.26 mmol/L ≤ TG<9.03 mmol/L**										
LDL-C(F)	9458	0.9145	0.8479	0.7072	0.6999 - 0.7143	9965	0.9336	0.8189	0.7419	0.7355 - 0.7482
LDL-C(M)	9458	0.9600	0.3114	0.9374	0.9351 - 0.9397	9965	0.9744	0.3662	0.9282	0.9258 - 0.9305
LDL-C(S)	9458	0.9486	0.5231	0.8481	0.8434 - 0.8526	9965	0.9551	0.5763	0.8424	0.8378 - 0.8468
LDL-C(NN)	9458	0.9581	0.2624	0.9523	0.9505 - 0.9541	9965	0.9807	0.1927	0.9788	0.9780 - 0.9796

**9.03 mmol/L ≤ TG<13.56 mmol/L**										
LDL-C(F)	180	0.8122	2.6938	0.3320	0.2745 - 0.3872	182	0.8646	2.5066	0.2808	0.2327 - 0.3275
LDL-C(M)	180	0.8486	1.0749	0.7278	0.6642 - 0.7809	182	0.8468	0.9853	0.6605	0.5933 - 0.7185
LDL-C(S)	180	0.7905	1.6666	0.4176	0.3505 - 0.4804	182	0.8745	1.5100	0.4037	0.3443 - 0.4599
LDL-C(NN)	180	0.8971	0.6305	0.8750	0.8405 - 0.9024	182	0.8889	0.4835	0.8815	0.8447 - 0.9100

**TG≥13.56 mmol/L**										
LDL-C(F)	149	0.6097	5.1902	0.0924	0.0652 - 0.1193	245	0.2440	7.1172	0.0624	0.0297 - 0.0950
LDL-C(M)	149	0.6394	2.8658	0.2302	0.1715 - 0.2872	245	0.4587	4.1364	0.2196	0.1603 - 0.2774
LDL-C(S)	149	0.5316	2.4093	0.0942	0.0631 - 0.1251	245	0.1155	4.4292	−0.0634	−0.1321 - 0.0058
LDL-C(NN)	149	0.7284	1.1388	0.6241	0.5437 - 0.6931	245	0.7024	2.1328	0.5958	0.5313 - 0.6534

TG, triglyceride; LDL-C(F), calculated LDL-C using the Friedewald formula; LDL-C(M), calculated LDL-C using the Martin/Hopkins formula; LDL-C(S), calculated LDL-C using the Sampson formula; LDL-C(NN), calculated LDL-C using the neural network-based AI formula; r, correlation coefficient; RMSE, root mean square error; CCC, Concordance Correlation Coefficient.

SI Conversion Factors: To convert LDL-C from mg/dL to mmol/L, multiply by 0.0259; to convert TG from mg/dL to mmol/L, multiply by 0.011.

**Fig. 2:**
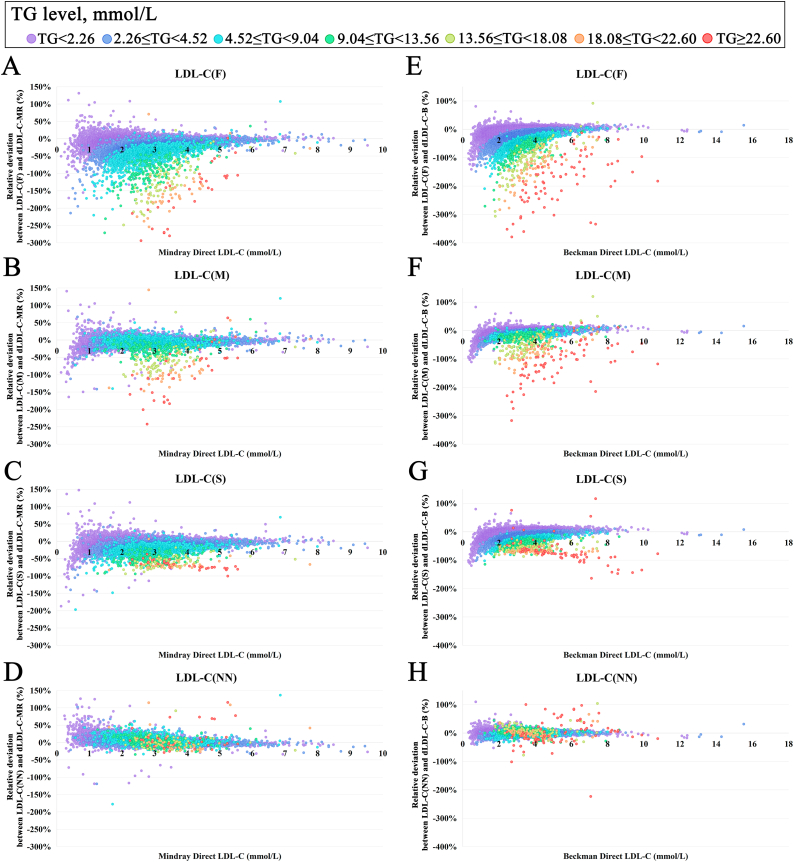
**Relative deviation of different calculated-LDL-C values with measured LDL-C values.** Relative deviation graph was plotted using the directly measured LDL-C(mmol/L) as the x-axis and the relative deviation between calculated and directly measured LDL-C as the y-axis. Figure A–D and E-H were using the directly measured LDL-C results from Mindray system and Beckman system as the x-axis. LDL-C: low-density lipoprotein cholesterol; LDL-C(F): calculated LDL-C using the Friedewald formula; LDL-C(M): calculated LDL-C using the Martin/Hopkins formula; LDL-C(S): calculated LDL-C using the Sampson equation; LDL-C(NN): calculated LDL-C using a neural network-based AI formula; dLDL-C-MR: Mindray directly measured LDL-C; dLDL-C-B: Beckman directly measured LDL-C. Individual dots on the graph representing individual samples are colored according to their triglyceride (TG) levels. Unit conversion factor for LDL-C, dLDL-C-MR, dLDL-C-B: mmol/L = 0.0259 × mg/dL.

In the Mindray system, LDL-C(F) exhibited 0.48 % negative results, while LDL-C(M) (0.08 %), LDL-C(S) (0.03 %), and LDL-C(NN) (0.01 %) demonstrated marked improvement in this phenomenon (Fig. 2). This pattern was also observed in the Beckman system [LDL-C(F): 0.34 % vs LDL-C(M): 0.08 % vs LDL-C(S): 0.03 % vs LDL-C(NN): 0.01 %].

### 3.3 Performance at different triglyceride levels

When TG levels were <2.26 mmol/L, all formulas demonstrated good concordance (CCC >0.95) between calculated LDL-C and directly measured LDL-C. In the subgroup with TG levels between 2.26 and 9.03 mmol/L, LDL-C(F) showed a modest decline in concordance with directly measured LDL-C (Mindray: CCC = 0.9145; Beckman: CCC = 0.9336), while other formulas maintained high concordance (CCC>0.95). At TG ≥ 9.03 mmol/L, all formulas exhibited reduced concordance with greater variability (Table 2), though LDL-C(NN) demonstrated the least variability among conventional formulas(for 9.03 mmol/L ≤ TG < 13.56 mmol/L: Mindray system - RMSE = 0.6305 mmol/L vs other formulas' RMSE = 1.0749–2.6938 mmol/L; Beckman system - RMSE = 0.4835 mmol/L vs other formulas' RMSE = 0.9853–2.5066 mmol/L).

**Fig. 3:**
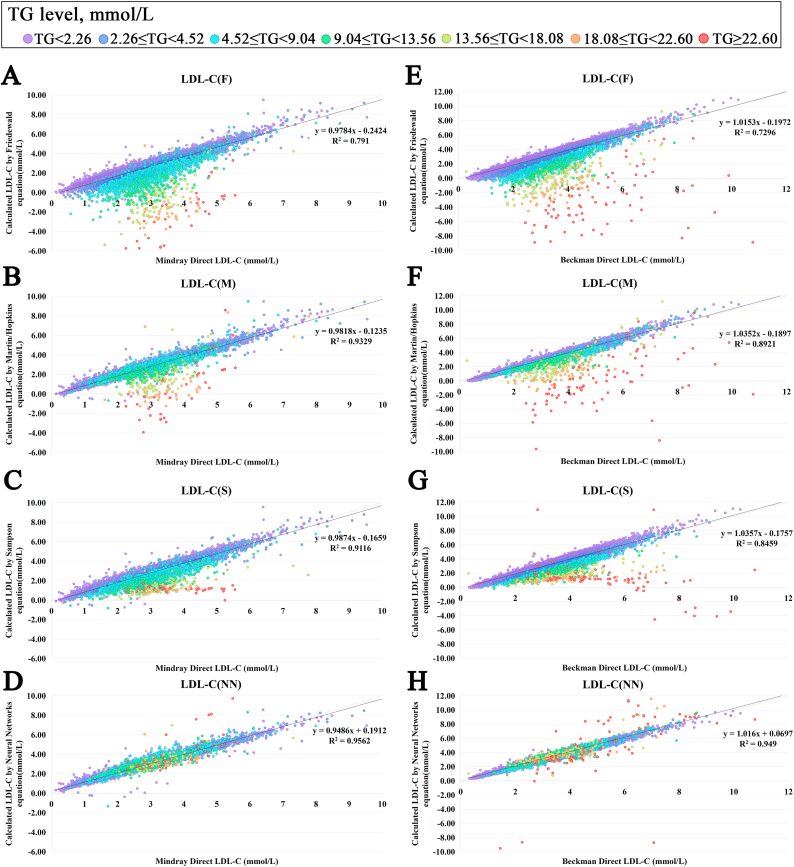
**Correlation of different calculated LDL-C values with measured LDL-C values.** Relative deviation graph was plotted using the directly measured LDL-C(mmol/L) as the x-axis and the relative deviation between calculated and directly measured LDL-C as the y-axis. Figure A–D and E-H were using the directly measured LDL-C results from Mindray system and Beckman system as the x-axis. LDL-C: low-density lipoprotein cholesterol; LDL-C(F): calculated LDL-C using the Friedewald formula; LDL-C(M): calculated LDL-C using the Martin/Hopkins formula; LDL-C(S): calculated LDL-C using the Sampson equation; LDL-C(NN): calculated LDL-C using a neural network-based AI formula. Individual dots on the graph representing individual samples are colored according to their triglyceride (TG) levels. The dashed line represents the identity line, and the solid line represents the linear fit of the regression equation. R^2^: coefficient of determination. Unit conversion factor for LDL-C, dLDL-C-MR, dLDL-C-B: mmol/L = 0.0259 × mg/dL.

Additionally, Fig. 3 indicated that the traditional formulas exhibited marked negative biases in high-TG ranges, while the average difference between predicted and measured LDL-C for LDL-C(NN) remained comparatively stable. When TG levels reached ≥9.03 mmol/L, the Mindray system showed LDL-C(NN) achieving the smallest mean bias compared to directly measured LDL-C [LDL-C(NN): 3.13 % vs LDL-C(F): 115.98 % vs LDL-C(M): 46.56 % vs LDL-C(S): 54.83 %]. This comparative advantage was consistently maintained in the Beckman system [LDL-C(NN): 1.14 % vs LDL-C(F): 120.50 % vs LDL-C(M): 54.08 % vs LDL-C(S): 44.32 %].

### 3.4 Clinical risk stratification accuracy

**Fig. 4:**
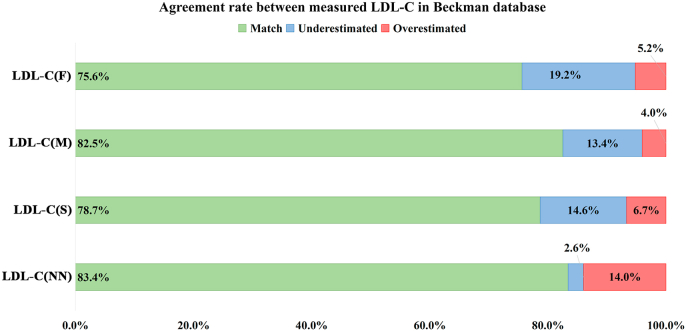
**Comparison of the clinical classification agreement rates between different calculated LDL-C and directly measured LDL-C in the Beckman database.** The clinical agreement rate is used as the x-axis, and different LDL-C calculation formulas are displayed along the y-axis. This figure calculates the clinical classification agreement rates between different LDL-C calculation formulas and the directly measured LDL-C results from Beckman. The green bars represent the percentage of patients correctly predicted, the blue bars represent the percentage of patients whose levels were underestimated, and the red bars represent the percentage of patients whose levels were overestimated. LDL-C: low-density lipoprotein cholesterol; LDL-C(F): calculated LDL-C using the Friedewald formula; LDL-C(M): calculated LDL-C using the Martin/Hopkins formula; LDL-C(S): calculated LDL-C using the Sampson equation; LDL-C(NN): calculated LDL-C using a neural network-based AI formula.

In the Mindray system, LDL-C(NN) achieved 87.5 % overall concordance, surpassing the Friedewald (66.6 %), Martin/Hopkins (77.0 %), and Sampson (74.2 %) formulas. This improvement translated to a 63 % reduction in patient misclassification into incorrect LDL-C treatment categories compared to the Friedewald formula. In the Beckman system, LDL-C(NN) achieved 83.4 % accuracy—comparable to the Martin/Hopkins method (82.5 %)—while outperforming both Friedewald (75.6 %) and Sampson (78.7 %) formulas, corresponding to a 32 % reduction in therapeutic misclassification rates relative to the Friedewald approach(Fig. 4).

**Fig. 5:**
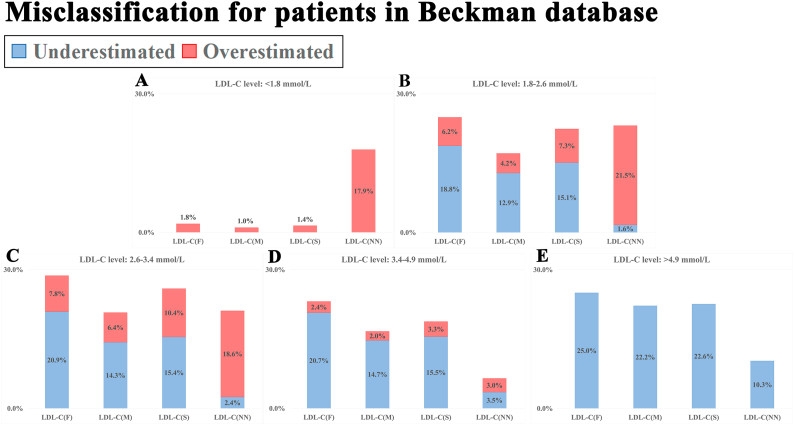
**Misclassification for patients in the Beckman database.** Using standard LDL-C cutoffs (1.8, 2.6, 3.4, and 4.9 mmol/L) from the Chinese guidelines for lipid management (2023 edition), we assessed how accurately each formula classified patients into clinical risk categories. Different LDL-C calculation formulas are plotted along the x-axis, and the misclassification rate is displayed on the y-axis. The blue bars represent the percentage of patients whose LDL-C levels were underestimated, while the red bars represent the percentage of patients whose levels were overestimated. LDL-C: low-density lipoprotein cholesterol; LDL-C(F): calculated LDL-C using the Friedewald formula; LDL-C(M): calculated LDL-C using the Martin/Hopkins formula; LDL-C(S): calculated LDL-C using the Sampson equation; LDL-C(NN): calculated LDL-C using a neural network-based AI formula. (A) LDL-C levels: <1.8 mmol/L; (B) LDL-C levels: 1.8–2.6 mmol/L; (C) LDL-C levels: 2.6–3.4 mmol/L; (D) LDL-C levels: 3.4–4.9 mmol/L; (E) LDL-C levels: >4.9 mmol/L.

Fig. 5 presents the accuracy of each formula in classifying patients according to clinically relevant LDL-C cutoffs (1.8, 2.6, 3.4, and 4.9 mmol/L). Further subgroup analysis revealed that the traditional formulas tended to underestimate LDL-C. In the Beckman system, When LDL-C levels fell within 3.4–4.9 mmol/L, traditional formulas resulted in underestimation for 14.7 %–20.7 % of cases, whereas LDL-C(NN) demonstrated significant improvement in this bias (3.5 %). However, LDL-C(NN) demonstrated higher rates of overestimation relative to other traditional formulas, especially when LDL-C levels less than 1.8 mmol/L (neural network Model: 17.9 %, vs Friedewald quation: 1.8 %; vs Martin/Hopkins quation: 1.0 %; vs Sampson quation: 1.4 %).

## 4 Discussion

This large-scale study (n = 188,887) is among the first to systematically apply a neural network algorithm to LDL-C estimation while evaluating the real-world clinical performance of established conventional formulas in Chinese populations [10]. Our findings indicate that the neural network model [LDL-C(NN)] substantially outperforms traditional formulas (Friedewald [5], Martin/Hopkins [7], Sampson [8]) in various triglyceride (TG) strata, particularly in cases of extreme hypertriglyceridemia (≥9.03 mmol/L), achieving a root mean square error (RMSE) as low as 0.4835–0.6305 mmol/L without producing biologically implausible negative values. Moreover, the clinical classification accuracy for LDL-C(NN) reached 87.5 % (Mindray) and 83.4 % (Beckman), surpassing that of other formulas (66.6–78.7 %). Importantly, LDL-C concentration plays a pivotal role in therapeutic decision-making, including patient risk stratification and selection of treatment modalities (e.g., lifestyle modification or pharmacological intervention). Underestimated LDL-C levels may lead to undertreatment of at-risk populations, consequently increasing cardiovascular disease risk. By significantly reducing LDL-C underestimation, LDL-C(NN) demonstrates potential to enhance atherosclerotic cardiovascular disease (ASCVD) risk prediction and optimize clinical management strategies.

The 2021 NLA Scientific Statement [16] recommends: For patients with LDL-C >2.6 mmol/L and TG ≤ 1.70 mmol/L, the Friedewald formula remains preferred, while cautioning against using LDL-C estimation equations when TG ≥ 4.52 mmol/L. Since its proposal in 1972, the Friedewald formula has remained in widespread clinical use. This equation assumes a fixed TG-to-VLDL-C ratio of 2.2:1, but becomes increasingly inaccurate when TG > 2.82 mmol/L and is explicitly contraindicated for TG ≥ 4.52 mmol/L [9]. Subsequent modifications by Vujovic et al. [18] adjusted this ratio to 3:1, while DeLong et al. [19] proposed a fixed coefficient of 2.7. Traditional equations rely on linear assumptions that often fail in hypertriglyceridemia, where an abundance of triglyceride-rich remnants (e.g., very low-density lipoprotein, intermediate-density lipoprotein) alters lipid composition. A fixed coefficient cannot fully capture this variability. Although Martin/Hopkins and Sampson formulas use dynamic or polynomial factors [7,8], they remain predominantly linear. While recent studies have explored machine learning for LDL-C estimation [[20], [21], [22]], there remains a notable absence of large-scale investigations applying neural network algorithms to LDL-C calculation in Chinese populations. Neural networks inherently learn complex nonlinear interactions among TC, TG, and HDL-C, enabling adaptive weighting of lipoprotein subfractions under elevated TG conditions. This approach achieves superior robustness compared to traditional methods, as evidenced by the significantly lower RMSE (0.4835–0.6305 vs 0.9853–2.6938 mmol/L) and higher CCC (0.8750–0.8815 vs 0.2808–0.7278) at TG ≥ 9.03 mmol/L in our multicenter validation.

Our analysis further demonstrates that LDL-C(NN) maintains good concordance (r > 0.97) on both the Mindray and Beckman systems. By merging data from two different analyzers and applying a stratified sampling method, the network effectively “learned” system-specific characteristics. Consequently, minimal additional training may suffice when transferring this model to other common clinical analyzers (e.g., Roche, Abbott). If an analyzer exhibits substantial bias, calibrating the network with a small local dataset could facilitate quick adaptation.

A “black box” perception is often cited as a limitation of neural networks [23,24]. To bolster clinical adoption, future research may employ interpretability tools [e.g., SHapley Additive exPlanations (SHAP), Local Interpretable Model-Agnostic Explanations (LIME)] to illustrate the relative importance of each input variable (TC, TG, HDL-C). Although full architectural transparency is unnecessary for everyday clinical use, insight into the main drivers of LDL-C prediction can heighten practitioners’ confidence in neural network outputs.

Several limitations should be considered in this study. One limitation is that formula accuracy was validated using homogeneous assays—clinically routine methods for direct LDL-C measurement—instead of ultracentrifugation or beta-quantification (the gold standard for LDL-C quantification). The direct assays employed calibration materials traceable to the Centers for Disease Control and Prevention (CDC) reference method for LDL-C, with satisfactory performance in National Health Commission external quality assessments (EQA) and accuracy verification programs. For hypertriglyceridemic samples, laboratories implemented instrument-based automatic dilution (with pre-validated maximum dilution factors) to ensure LDL-C result accuracy. Consequently, the concordance between formula-derived and directly measured LDL-C provides a valid preliminary assessment of formula performance in Chinese populations. The other is that our neural network model was developed using lipid profile measurements from a single-center dataset at Peking University Shenzhen Hospital. Although internal validation was performed, further external validation is required to confirm the model's generalizability and accuracy. Additionally, this study focused on developing and validating the model's accuracy across different LDL-C and TG levels, and did not analyze the impact of various patient clinical characteristics (e.g., race, kidney disease, diabetes, use of lipid-lowering medications) on model performance. Finally, while the developed model is not a simple linear formula and requires computational processing by physicians, we have saved and exported it as a simplified formula executable in spreadsheet software (e.g., Microsoft Excel). This approach simplifies the model's application and can help mitigate this limitation to some extent.

Future work should collect data from a wider range of patient populations, including different geographic and ethnic groups, to strengthen external validation. A multicenter study design would also further confirm the generalizability of these findings. Additionally, the neural network model developed in this study holds potential to guide laboratory autoverification processes and provide actionable references when the sum of directly measured LDL-C and HDL-C exceeds total cholesterol (TC). Future research should explore expanded clinical application scenarios for LDL-C calculation formulas, particularly in reconciling discordant lipid profiles and optimizing ASCVD risk management protocols. Moreover, in 2024, the National Lipid Association (NLA) issued an expert consensus that highlighted the role of apolipoprotein B (ApoB) in the clinical management of cardiovascular risk in adults [[23], [24], [25]], the value of which has been increasingly recognized by experts and researchers. However, direct measurement of ApoB has certain limitations, such as issues with standardization and relatively high cost. Consequently, researchers have developed calculation-based methods to estimate ApoB levels. In a recent large-scale cohort of Southern European subjects, Cicero et al. evaluated the reliability of 23 ApoB estimation formulas, observing a maximum concordance of only 41 %. This finding highlights the necessity for further refinement in developing ApoB estimation methods for clinical application [23,24,26]. Therefore, future studies could focus on developing ApoB estimation formulas specifically tailored for Chinese populations.

## 5 Conclusion

In this study involving two analytical platforms and over 180,000 lipid profiles, we comprehensively assessed the performance of Friedewald, Martin/Hopkins, and Sampson formulas and developed a novel neural network–based model [LDL-C(NN)] for LDL-C estimation. By leveraging nonlinear feature learning, the model successfully circumvents the limitations of conventional linear formulas, particularly in hypertriglyceridemia, and avoids negative or biologically inconsistent outputs. Additional data from multi-ethnic cohorts with very low LDL-C or rare dyslipidemias will be crucial for confirming and extending the model's clinical utility.

## Funding

This work was supported by 10.13039/501100015956Special Project for Research and Development in Key areas of Guangdong Province (2020B1111160001) and Wu Jieping Medical Foundation (320.6750.2025-6-6). The funders had no role in study design, data collection and analysis, decision to publish, or preparation of the manuscript.

## Declaration of competing interest

The authors declare the following financial interests/personal relationships which may be considered as potential competing interests:Youlin Liu reports financial support was provided by Special Project for Research and Development in Key areas of Guangdong Province and Hao Xue reports financial support was provided by Wu Jieping Medical Foundation. Reports a relationship with that includes:. Has patent pending to. If there are other authors, they declare that they have no known competing financial interests or personal relationships that could have appeared to influence the work reported in this paper.

## Figures and Tables

**figs1:**
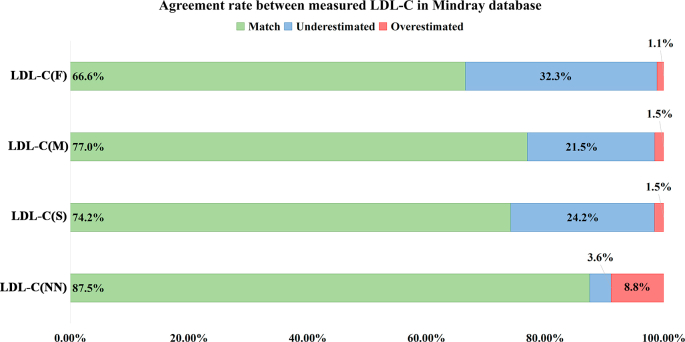


**figs2:**